# An 18-Year Follow-up Survey of Dioxin Levels in Human Milk in Japan

**DOI:** 10.2188/jea.JE20170032

**Published:** 2018-06-05

**Authors:** Ryusuke Ae, Yosikazu Nakamura, Hiroshi Tada, Yumi Kono, Eiko Matsui, Kazuo Itabashi, Masanori Ogawa, Teppei Sasahara, Yuri Matsubara, Takao Kojo, Kazuhiko Kotani, Nobuko Makino, Yasuko Aoyama, Takashi Sano, Koki Kosami, Maho Yamashita, Akira Oka

**Affiliations:** 1 Division of Public Health, Center for Community Medicine, Jichi Medical University, Tochigi, Japan; 2 Health Service Center, Jichi Medical University, Tochigi, Japan; 3 Department of Neonatology, Toho University School of Medicine, Tokyo, Japan; 4 Department of Pediatrics, Jichi Medical University, Tochigi, Japan; 5 Department of Pediatrics, Graduate School of Medicine, Gifu University, Gifu, Japan; 6 Department of Pediatrics, Showa University of Medicine, Tokyo, Japan; 7 Department of Pediatrics, Tokyo University, Tokyo, Japan

**Keywords:** dioxins, polychlorinated biphenyls, human milk, epidemiology, monitoring

## Abstract

**Background:**

Globally, few published studies have tracked the temporal trend of dioxin levels in the human body since 2000. This study describes the annual trend of dioxin levels in human breast milk in Japanese mothers from 1998 through 2015.

**Methods:**

An observational study was conducted from 1998 through 2015. Participants were 1,194 healthy mothers following their first delivery who were recruited annually in Japan. Breast milk samples obtained from participants were analyzed using gas chromatography and mass spectrometry for dioxins, including polychlorinated dibenzo-p-dioxins (PCDDs), polychlorinated dibenzofurans (PCDFs), and coplanar polychlorinated biphenyls (PCBs).

**Results:**

Mean age was 29.5 years, and 53% of participants were 20–25 years old. A declining trend in total dioxin levels was found, from a peak of 20.8 pg toxic equivalence (TEQ)/g fat in 1998 to 7.2 pg TEQ/g fat in 2014. Data from the last 5 years of the study indicated a plateau at minimal levels. In contrast, an increasing trend was found in the mean age of participants during the last 5 years. Although significantly higher dioxin levels were observed in samples from older participants, an upward trend in dioxin levels was not observed, indicating that dietary and environmental exposure to dioxins had greatly diminished in recent years.

**Conclusions:**

Dioxin levels in human breast milk may be approaching a minimum in recent years in Japan. The findings may contribute to global reference levels for environmental pollution of dioxins, which remains a problem for many developing countries.

## INTRODUCTION

Dioxins are environmental pollutants, which are of concern because their toxic potentials affect the health of humans and other species. Dioxins are not primary products but non-intentional byproducts derived from various processes, such as industrial manufacturing, chemical synthesis, or waste incineration.^1^^–^^3^ They can also result from natural processes, such as volcanic eruptions and forest fires.^3^ The process of dioxin production is still only partially understood.

Dioxins include three main categories of chemically-related compounds—polychlorinated dibenzo-*p*-dioxins (PCDDs), polychlorinated dibenzofurans (PCDFs), and dioxin-like polychlorinated biphenyls (PCBs)—and cause various problems, including reproductive and developmental disorders,^4^^–^^8^ interference with endocrine systems,^4^^–^^6^ and tumor promotion.^9^^–^^13^ Because these effects are species-specific and there is little definitive evidence, long-term effects of dioxin toxicity on human health remain controversial.^2^^,^^8^^,^^9^^,^^14^^,^^15^

Dioxin levels in breast milk reflect dietary and environmental exposure, as well as neonatal exposure.^2^^,^^16^^,^^17^ Because dioxin levels in multiparous mothers are significantly lower than those in primiparous mothers,^17^^–^^20^ dioxins are thought to be transferred from mother to infant via breastfeeding. We previously reported that dioxin levels in breast milk declined in the general Japanese population between 1997 and 2002.^18^ We have continued to monitor dioxin levels in breast milk. Globally, some studies have been designed only to compare the latest dioxin levels in human milk samples with levels recorded in the single previous year. Few studies have assessed annual follow-up trends in dioxin levels in human milk. Actually, no published study has tracked the temporal annual trend of dioxins over the past decade since 2000, except for surveys conducted in Sweden.^21^^–^^23^ Here, we describe the annual trend of dioxins levels in breast milk among healthy Japanese mothers between 1998 and 2015.

## METHODS

### Settings and participants

**Figure 1.**
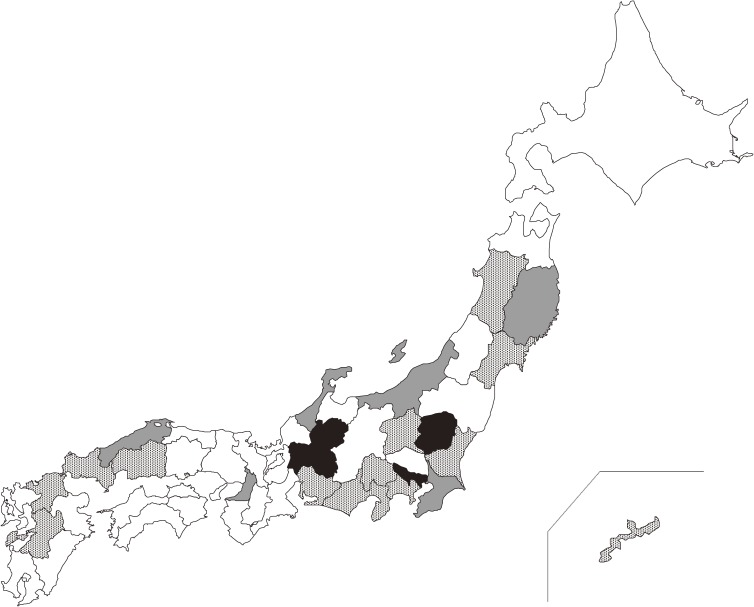
Sampling of the study participants: A map of Japanese prefectures. Dots represent 13 prefectures that participated in 1998 (Miyagi, Akita, Ibaraki, Gunma, Kanagawa, Yamanashi, Shizuoka, Aichi, Hiroshima, Yamaguchi, Fukuoka, Kumamoto, and Okinawa). Gray represents six prefectures that participated during the period 1998–2010 (Iwate, Chiba, Niigata, Ishikawa, Osaka, and Shimane). Black represents three prefectures that participated during the period 2010–2015 (Tochigi, Tokyo, and Gifu).

Eligible participants were 1,194 healthy mothers following their first delivery who were recruited annually from 19 prefectures in 1998 (Iwate, Miyagi, Akita, Ibaraki, Gunma, Chiba, Kanagawa, Niigata, Ishikawa, Yamanashi, Shizuoka, Aichi, Osaka, Shimane, Hiroshima, Yamaguchi, Fukuoka, Kumamoto, and Okinawa), six prefectures during 1999–2009 (Iwate, Chiba, Niigata, Ishikawa, Osaka, and Shimane), and three prefectures during 2010–2015 (Tochigi, Tokyo, and Gifu) (Figure 1). We show sample sizes of participants with the mean age by sampling year and prefecture in eTable 1. Women who delivered for the second or third time were not enrolled because dioxin levels in their breast milk were presumed to have declined with previous breastfeeding.^17^^–^^20^ We obtained approximately 30–50 mL of breast milk, which was manually squeezed by each participant at the 30th day after delivery. All participants provided signed informed consent. The study was approved by the bioethics committee of Jichi Medical University and Tokyo University.

### Measurements

Demographic characteristics included age, height, weight, and body mass index (BMI). Age was classified into three age groups (20–25, 26–30, and ≥31 years).

Measurement methods for dioxins were the same as in our previous report.^18^ PCDDs (seven isomers), PCDFs (10 isomers), and coplanar PCBs (12 isomers) were analyzed using gas chromatography and mass spectrometry. Milk samples were mixed with an aqueous solution of sodium oxalate, diethyl ether, and ethanol, and the mixture was extracted with hexane. A three-step clean-up procedure was performed using a column filled with silica gel, followed by another column containing aluminum oxide, then by an activated charcoal column. After concentrating the sample, gas chromatography and mass spectrometry were used to measure the contents. Dioxin was quantified as the toxic equivalence (TEQ) value per gram of fat in breast milk. TEQ was calculated with toxic equivalent factors of 2,3,7,8-tetrachlorodibenzodioxin, which was reported in the 2005 World Health Organization re-evaluation of toxic equivalency for dioxins.^24^ Dioxin levels lower than the lower detection limit of quantitation were calculated as zero. Total dioxins were defined as the sum of PCDDs, PCDFs, and coplanar PCBs.

Dioxin measurements were outsourced annually and were measured at four individual laboratories that were certificated for measurement quality by the Japanese government: Japan Food Research Laboratory (1998–2006); SRL, Inc. (2007–2012); Otsuka Pharmaceutical Co., Ltd. (2013–2014); and Kitakyushu Life Science Center (2015). Each year that the laboratory changed, we asked both the new and old laboratories to conduct testing (double-checking) so we could compare laboratory results for consistency.

### Statistical analysis

The three age groups are presented as percentages of the study population, and other numerical variables are presented as the means with standard deviations (SDs), medians, minimums, and maximums. Dioxin levels of all samples are presented as the cohort mean (*n* = 1,194) with 95% confidence intervals instead of SDs. We described the total dioxin level trends for 1998–2015 with the means and 95% confidence intervals, as well as those of PCDDs, PCDFs, and coplanar PCBs with the means. Trends in the mean age of participants for 1998–2015 were also described. Differences in dioxin levels among the three age groups were compared using analysis of covariance (ANCOVA) testing with the significance threshold set at *P* < 0.05, adjusting for sampling year, prefecture, and BMI. All analyses were performed using IBM SPSS Statistics for Windows, Version 23 (IBM Corp., Armonk, NY, USA).

## RESULTS

Table 1 lists demographic characteristics of the study sample (*n* = 1,194). The mean was 29.5 (SD, 3.1) years old; the proportion of the three age groups (20–25, 26–30, and ≥31 years) were 10%, 53%, and 37%, respectively. Table 2 shows dioxin levels in breast milk of all samples for 1998–2015. The means for total dioxins, PCDDs, PCDFs, and coplanar PCBs were 17.0, 7.9, 3.0, and 6.1 pg TEQ/g fat, respectively. Dioxin levels for each congener are shown in eTable 2.

**Table 1.** Demographic characteristics of mothers (*n* = 1,194)

	Mean (SD)		Median	(Min–Max)
Age, years	29.5 (3.1)		29	(20–45)
20–25	*n* = 113	(10%)		
26–30	*n* = 637	(53%)		
≥31	*n* = 444	(37%)		

Height, cm	158.1 (5.4)		158.0	(140–175)
Weight, kg	52.1 (7.7)		51.0	(37.5–96.0)
BMI, kg/m^2^	20.8 (2.7)		20.3	(16.0–36.2)

BMI, body mass index; Max, maximum; Min, minimum; SD, standard deviation.

**Table 2.** Dioxin levels in human breast milk for all samples, 1998–2015 (*n* = 1,194)

Dioxin Levels (pg TEQ/g fat)^a^	Mean	(95% CI)	Median	(Min–Max)
Total dioxins^b^	17.00	(16.58–17.42)	16.18	(2.09–47.09)
PCDDs (7 isomers)	7.92	(7.71–8.13)	7.54	(1.01–25.4)
PCDFs (10 isomers)	3.03	(2.95–3.12)	2.82	(0.53–15.76)
Coplanar-PCBs (12 isomers)	6.05	(5.88–6.23)	5.48	(0.07–35.19)

CI, confidence interval; Max, maximum; Min, minimum; PCB, polychlorinated biphenyl; PCDD, polychlorinated dibenzo-*p*-dioxin; PCDF, polychlorinated dibenzofuran; TEQ, toxic equivalence.

^a^Dioxin levels are presented as the WHO-TEQ (2005); values lower than the lower limit of quantitation were calculated as zero.

^b^Total dioxins = PCDDs (7 isomers) + PCDFs (10 isomers) + coplanar PCBs (12 isomers).

**Figure 2.**
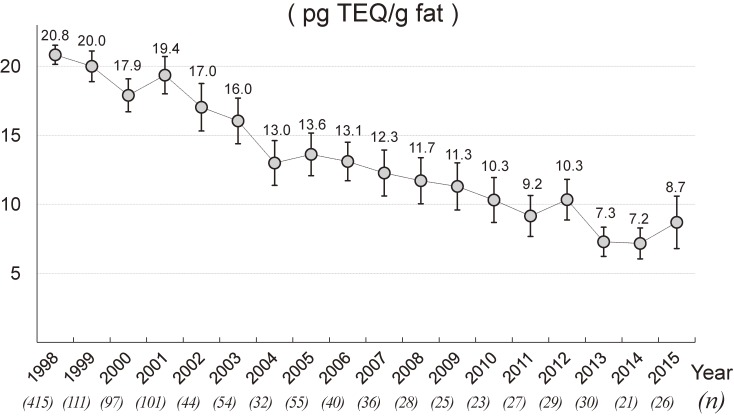
Annual trends in levels of total dioxins, 1998–2015. Total dioxins = PCDDs (7 isomers) + PCDFs (10 isomers) + coplanar PCBs (12 isomers). Annual levels of the total dioxins are shown with the means and 95% confidence intervals during the study period (1998–2015). Each value given in parentheses under the calendar year represents the sample size in each year.

**Figure 3.**
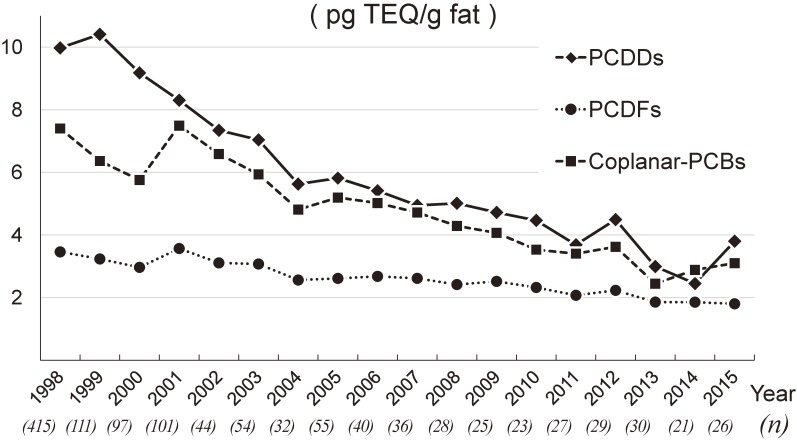
Annual trends in levels of PCDDS, PCDFs, and coplanar PCBs, 1998–2015. Annual levels of PCDDs (7 isomers), PCDFs (10 isomers), and coplanar PCBs (12 isomers) are shown with the means during the study period (1998–2015). Each value given in parentheses under the calendar year represents the sample size in each year. The 95% confidence interval bars were omitted for figure clarity. PCDD, polychlorinated dibenzo-*p*-dioxin; PCDF, polychlorinated dibenzofuran; PCB, polychlorinated biphenyl.

Figure 2 illustrates annual trends in total dioxin levels for 1998–2015. Total dioxin levels tended to decline over time from a peak of 20.8 pg TEQ/g fat in 1998 to a low of 7.2 pg TEQ/g fat in 2014. Additively, these trends indicate a plateau in the last 5 years. Individual levels for PCDDs, PCDFs, and coplanar PCBs also followed this trend (Figure 3).

**Figure 4.**
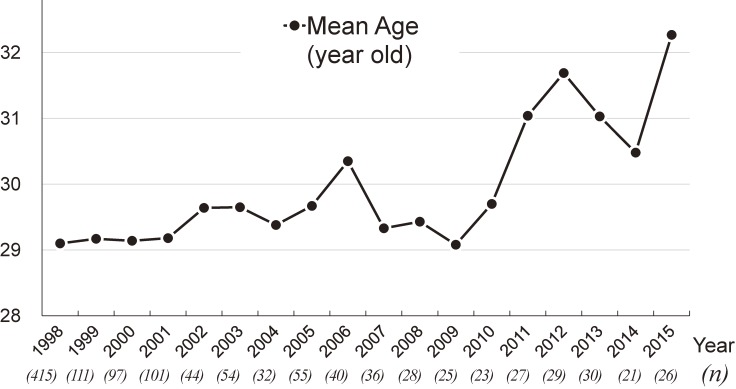
Annual trends in the mean age of participants, 1998–2015. The mean ages of participants are shown for each year of the study period (1998–2015). Each value given in parentheses under the calendar year represents the sample size in each year.

**Figure 5.**
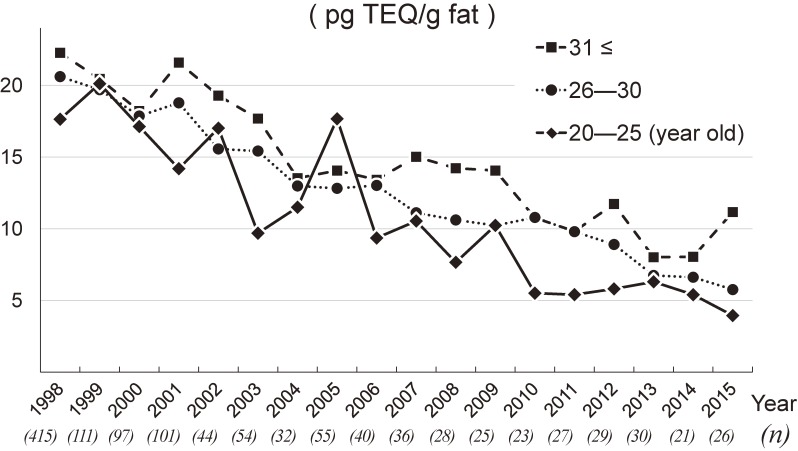
Annual trends in levels of total dioxins among three age groups, 1998–2015. Total dioxins, PCDDs (7 isomers) + PCDFs (10 isomers) + coplanar PCBs (12 isomers). Annual levels of total dioxins among three age groups are shown with the means during the study period (1998–2015). Each value given in parentheses under the calendar year represents the sample size in each year.

Figure 4 shows the temporal trend in the mean age of participants during the study period. In contrast to dioxin levels, the mean age increased in the last 5 years, with a mean age above 30 years in each year since 2011. Table 3 shows dioxin levels among the three age groups. Total dioxin levels were higher in the higher-age groups, indicating that dioxins accumulate with age. These differences were statistically significant, even after adjusting for sampling year, prefecture, and BMI (*P* < 0.01, ANCOVA). Figure 5 shows annual levels of total dioxins among the three age groups, represented with the means during the study period (1998–2015). Although higher dioxin levels were observed in the older groups, the mean levels for total dioxins indicated similar declining trends in all three age groups.

**Table 3.** Dioxin levels among three age groups (*n* = 1,194)

Dioxin Levels (pg TEQ/g fat)^b^		Age group, years						*P*-value^a^	
	
		20–25		26–30		≥31		Crude	Adjusted^c^
		(*n* = 113)		(*n* = 637)		(*n* = 444)			
Total dioxins^d^	Mean (95% CI)	15.0	(13.7–16.3)	16.8	(16.2–17.3)	17.8	(17.1–18.5)	<0.01	<0.01
PCDDs	Mean (95% CI)	7.1	(6.4–7.7)	7.8	(7.5–8.1)	8.3	(8.0–8.7)	<0.01	<0.01
PCDFs	Mean (95% CI)	2.7	(2.5–2.9)	3.0	(2.9–3.1)	3.2	(3.1–3.3)	<0.01	<0.01
Coplanar-PCBs	Mean (95% CI)	5.2	(4.7–5.8)	6.0	(5.8–6.3)	6.3	(6.0–6.6)	<0.01	<0.01

CI, confidence interval; PCB, polychlorinated biphenyl; PCDD, polychlorinated dibenzo-*p*-dioxin; PCDF, polychlorinated dibenzofuran; TEQ, toxic equivalence.

^a^Analysis of covariance testing.

^b^Dioxin levels are presented as the WHO-TEQ (2005).

^c^Adjusted for sampling year, prefecture, and body mass index.

^d^Total dioxins = PCDDs (7 isomers) + PCDFs (10 isomers) + coplanar PCBs (12 isomers).

## DISCUSSION

This study is our second report, which follows our previously conducted human milk survey for dioxins in the Japanese general population during 1997–2002.^18^ In the current study, we found that total dioxin levels, including PCDDs, PCDFs, and coplanar PCBs, declined sequentially after 2002, and appear to have reached a plateau at minimal levels in 2011–2015. The mean age of mothers increased over the same period. Despite the finding that significantly higher dioxin levels were observed in older age groups, an upward trend in dioxin levels was not observed, indicating that dietary and environmental exposure to dioxins have diminished in recent years in Japan.

The recent study period included a higher percentage of older participants. In Japan and other developed countries, maternal age at first delivery has increased. Dioxin levels in breast milk accumulate with age,^14^^,^^25^^,^^26^ a finding that was confirmed in our study (Table 3). Although participants were generally older in the recent study period, our findings indicate that total dioxin levels did not increase but seemed to plateau. Furthermore, dioxin levels showed similar declining trends in all three age groups, which could reflect declining dioxin levels in the environment.

Lower dioxin levels in breast milk can reflect lower past dietary and environmental exposure. Of these exposures, dietary intake is the primary route of human exposure to dioxins.^27^^,^^28^ Specifically, fish and shellfish contain more dioxins than other foods.^14^^,^^29^^–^^31^ Japanese government publications report that fish and shellfish intake tended to decline over the past few decades among Japanese women^32^^,^^33^; this would support our findings that dioxin levels in breast milk declined over time. A reduction in environmental emissions of dioxins would be another reason. Since the Law Concerning Special Measures against Dioxins was introduced in Japan in 1999,^34^ environmental emissions of dioxins has decreased; consequently, environmental contamination of dioxins would decrease throughout Japan.

Studies from other countries have reported that dioxin levels in human milk have declined in recent years, with the latest mean concentrations reported as 8.3 pg TEQ/g fat in Canada,^35^ 17.8 pg TEQ/g fat in France,^36^ 11.3 pg TEQ/g fat in Hong Kong^37^ and 9.7 pg TEQ/g fat in Ireland.^38^ However, these studies^35^^–^^38^ only compared the latest dioxin levels in human milk samples with levels from the single previous year. No studies have assessed the temporal follow-up trend in dioxin levels in human milk in the same manner as the current study, except for surveys in Sweden (4.6 pg TEQ/g fat in 2011).^21^^–^^23^ In China, specifically, these levels were found to follow an upward trend from 2007 to 2012 (5.4 pg TEQ/g fat in 2007 and 8.3 pg TEQ/g fat in 2012).^39^ Lu et al^39^ concluded that the trend was associated with release of pollutants due to rapid industrialization and urbanization and suggested that monitoring dioxin levels is still required in rapidly growing countries. Our findings can serve as a reference value of dioxin levels for developing countries, because they indicate that Japan has reached a plateau at minimal levels in the past few years.

Lower dioxin levels in breast milk also reflect lower neonatal exposure via breastfeeding. Dioxins are transferred from mother to baby via breastfeeding.^17^^–^^20^ Our colleagues previously reported that no significant correlation was found between neonatal dioxin exposure via breastfeeding and children’s behavioral and psychosocial problems, as measured using an international standard scale, supporting the benefits of breastfeeding.^40^ Nevertheless, they also reported that daily dioxin intake of children via breastfeeding was estimated as 40–50 pg TEQ/kg/day, which is higher than the tolerable intake (1–4 pg TEQ/kg/day; 70 pg TEQ/kg/month) recommended by the WHO.^3^^,^^40^^,^^41^ Kono et al^40^ used the same participants and samples (mothers and their breast milk) as the current study, although additional participants were recruited for the current study. Children in the study^40^ were fed breast milk that contained mean total dioxin levels ranging from 13.0 through 20.8 pg TEQ/g fat in the period 1998–2005 (numbers derived by the current study). This range is higher than that of the past 5 years (7.2 to 10.3 pg TEQ/g fat). Actually, daily dioxin intake for children via breastfeeding at recent years is still higher than that recommended by the WHO.^3^^,^^41^ If dioxin levels remain at their current levels or decline further, fewer developmental effects should be seen in children. However, reproductive problems still cannot be evaluated. Little data are available on effects such as endometriosis or time-to-pregnancy.^5^^,^^42^ Further studies are needed to determine whether reproductive problems would manifest themselves when these children reach reproductive age.

There are three major limitations in our study. The primary limitation is participants’ residence and their lifestyles, particularly dietary habits. Our colleagues previously assessed the correlation between the distance of the place of residence from waste incinerators and concentrations of contaminants in milk.^43^ Participants (*n* = 240) in Tokyo (six districts) showed no correlations between dioxin levels in milk and distance from their home to the nearest waste incinerator.^43^ However, residing area could affect dietary habits, such as shellfish intake, and therefore affect dioxin exposure. According to the evidence from cardiovascular medicine, a study reported on differences in fish intake between fishing and farming communities in Japan and the resulting difference in the incidence of cardiovascular diseases.^44^ These findings suggest that dioxin exposure could be affected by residing area because of differences in dietary habits. Our study did not assess residence and dietary habits in detail, which might affect the results.

The second limitation is sampling prefectures and sample size. With accumulating evidence that suggests environmental exposure to dioxins is decreasing in Japan, research collaborators, volunteer participants, and government research funding have been gradually decreasing. Nevertheless, daily dioxin intake of children via breastfeeding may not achieve the tolerable level recommended by the WHO.^3^^,^^41^ Measuring dioxin levels in breast milk is costly, which also limits the feasible sample size. Accordingly, the sampling prefecture was changed twice, and only three prefectures were sampled after 2009, which are not included in our previous research.^18^ The study may no longer reflect the general Japanese population. However, no strong change in decreasing trend was observed over the two study periods, and dioxin levels also decreased. For this reason, the sampling process may not have had an impact on the results.

The third limitation is the quality of dioxin measurements. Milk samples were obtained and measured each year. The accuracy of analysis method for dioxin would have advanced over time. Dioxin levels determined during early study periods might differ (higher) with differing (advancing) sensitivity of analysis. Our results do, however, indicate a decreasing trend.

In conclusion, dioxin levels in human breast milk, including PCDDs, PCDFs, and coplanar PCBs, may approach a minimum in the near future in Japan. Our results may contribute as reference levels for global studies of dioxin contamination, which is still a major concern for many countries. Dioxin levels in human breast milk should still be monitored because the production mechanisms of dioxins are not completely known, and other sources of dioxins may emerge, although emissions have been fairly controlled in Japan.

## APPENDIX A. SUPPLEMENTARY DATA

eTable 1. Sample size of participants with the mean age by sampling year and prefecture (n = 1,194)

eTable 2. Dioxin levels of all isomers in human breast milk for all samples: 1998–2015 (n = 1,194)
