# Supplementary material for: An 18-Year Follow-up Survey of Dioxin Levels in Human Milk in Japan
Source: J Epidemiol. 2018 Jun 5;28(6):300–6. doi: 10.2188/jea.JE20170032 (PMC5976874; doi:10.2188/jea.JE20170032)
Supplement: Supplementary file 1 [file je-28-300-s001.pdf]

eTable 1. Sample size of participants with the mean age by sampling year and prefecture (n=1,194)

	Calendar Year																		
	**1998**	**1999**	**2000**	**2001**	**2002**	**2003**	**2004**	**2005**	**2006**	**2007**	**2008**	**2009**	**2010**	**2011**	**2012**	**2013**	**2014**	**2015**	**Total**
**Prefectures**																			
Iwate	29.5 (n=20)	29.5 (n=20)	29.9 (n=15)	29.8 (n=22)	29.8 (n=8)	30.2 (n=10)	30.3 (n=6)	30.8 (n=5)	31.0 (n=3)	—	—	—	—	—	—	—	—	—	30.1 (n=109)
Miyagi	29.0 (n=20)	—	—	—	—	—	—	—	—	—	—	—	—	—	—	—	—	—	29.0 (n=20)
Akita	28.9 (n=20)	—	—	—	—	—	—	—	—	—	—	—	—	—	—	—	—	—	28.9 (n=20)
Ibaraki	29.5 (n=20)	—	—	—	—	—	—	—	—	—	—	—	—	—	—	—	—	—	29.5 (n=20)
Tochigi	—	—	—	—	—	—	—	—	—	—	—	—	29.3 (n=9)	27.7 (n=6)	33.1 (n=9)	33.2 (n=10)	31.4 (n=7)	33.8 (n=9)	31.4 (n=50)
Gunma	29.6 (n=20)	—	—	—	—	—	—	—	—	—	—	—	—	—	—	—	—	—	29.6 (n=20)
Chiba	29.1 (n=20)	28.3 (n=20)	29.4 (n=20)	29.1 (n=20)	29.0 (n=7)	30.1 (n=9)	28.6 (n=11)	28.2 (n=10)	29.9 (n=13)	28.7 (n=12)	31.0 (n=2)	28.6 (n=5)	30.0 (n=1)	—	—	—	—	—	29.2 (n=150)
Tokyo	—	—	—	—	—	—	—	—	—	—	—	—	—	31.9 (n=17)	31.3 (n=13)	28.8 (n=10)	30.1 (n=7)	30.3 (n=7)	30.5 (n=54)
Kanagawa	29.1 (n=65)	—	—	—	—	—	—	—	—	—	—	—	—	—	—	—	—	—	29.1 (n=65)
Niigata	28.7 (n=20)	29.8 (n=20)	29.3 (n=20)	29.4 (n=20)	28.8 (n=12)	29.7 (n=15)	31.4 (n=5)	29.6 (n=18)	29.9 (n=9)	29.6 (n=13)	28.6 (n=14)	28.3 (n=8)	—	—	—	—	—	—	29.4 (n=174)
Ishikawa	29.4 (n=10)	28.9 (n=11)	28.6 (n=7)	28.1 (n=9)	31.7 (n=3)	30.0 (n=4)	30.0 (n=4)	28.9 (n=8)	30.5 (n=4)	—	—	—	—	—	—	—	—	—	29.6 (n=60)
Yamanash	28.9 (n=20)	—	—	—	—	—	—	—	—	—	—	—	—	—	—	—	—	—	28.9 (n=20)
Gifu	—	—	—	—	—	—	—	—	—	—	—	—	29.8 (n=5)	32.5 (n=4)	30.6 (n=7)	31.1 (n=10)	29.9 (n=7)	32.3 (n=10)	31 (n=43)
Shizuoka	29.2 (n=20)	—	—	—	—	—	—	—	—	—	—	—	—	—	—	—	—	—	29.2 (n=20)
Aichi	29.0 (n=20)	—	—	—	—	—	—	—	—	—	—	—	—	—	—	—	—	—	29.0 (n=20)
Osaka	28.6 (n=20)	29.3 (n=20)	28.7 (n=16)	28.6 (n=15)	29.9 (n=13)	28.9 (n=16)	27.7 (n=6)	30.9 (n=14)	31.1 (n=11)	29.7 (n=11)	30.2 (n=12)	29.8 (n=12)	30.0 (n=8)	—	—	—	—	—	29.5 (n=174)
Shimane	29.2 (n=20)	29.2 (n=20)	28.7 (n=19)	29.3 (n=15)	34.0 (n=1)	—	—	—	—	—	—	—	—	—	—	—	—	—	30.1 (n=75)

Hiroshima	28.8 (n=20)	—	—	—	—	—	—	—	—	—	—	—	—	—	—	—	—	—	28.8 (n=20)
Yamaguch	29.1 (n=20)	—	—	—	—	—	—	—	—	—	—	—	—	—	—	—	—	—	29.1 (n=20)
Fukuoka	29.3 (n=20)	—	—	—	—	—	—	—	—	—	—	—	—	—	—	—	—	—	29.3 (n=20)
Kumamoto	29.2 (n=20)	—	—	—	—	—	—	—	—	—	—	—	—	—	—	—	—	—	29.2 (n=20)
Okinawa	29.5 (n=20)	—	—	—	—	—	—	—	—	—	—	—	—	—	—	—	—	—	29.5 (n=20)
**Total**	29.1 (n=415)	29.2 (n=111)	29.1 (n=97)	29.2 (n=101)	29.6 (n=44)	29.7 (n=54)	29.4 (n=32)	29.7 (n=55)	30.4 (n=40)	29.3 (n=36)	29.4 (n=28)	29.1 (n=25)	29.7 (n=23)	31.0 (n=27)	31.7 (n=29)	31.0 (n=30)	30.5 (n=21)	32.3 (n=26)	30.0 (n=1,194)

eTable 2. Dioxin levels of all isomers in human breast milk for all samples: 1998–2015 (n=1,194)

**Dioxin Levels (pg TEQ/g fat)**[a]	Mean	(95% CI)	Median	(Min–Max)
**Total dioxins**[b]	17.00	(16.58 – 17.42)	16.18	(2.09 – 47.09)
**PCDD (7 isomers)**	7.92	(7.71 – 8.13)	7.54	(1.01 – 25.39)
2,3,7,8-TetraCDD	1.00	(0.97 – 1.04)	1.00	(0.00 – 5.00)
1,2,3,7,8-PentaCDD	4.67	(4.56 – 4.79)	4.40	(0.78 – 17.00)
1,2,3,4,7,8-HexaCDD	0.14	(0.14 – 0.15)	0.15	(0.00 – 0.91)
1,2,3,6,7,8-HexaCDD	1.73	(1.68 – 1.79)	1.60	(0.00 – 7.30)
1,2,3,7,8,9-HexaCDD	0.27	(0.25 – 0.28)	0.25	(0.00 – 1.30)
1,2,3,46,7,8-HeptaCDD	0.08	(0.08 – 0.08)	0.07	(0.00 – 0.49)
OctaCDD	0.02	(0.01 – 0.02)	0.01	(0.00 – 0.15)
**PCDF (10 isomers)**	3.03	(2.95 – 3.11)	2.82	(0.53 – 15.76)
2,3,7,8-TetraCDF	0.06	(0.05 – 0.06)	0.06	(0.00 – 1.50)
1,2,3,7,8-PentaCDF	0.00	(0.00 – 0.01)	0.00	(0.00 – 0.26)
2,3,4,7,8-PentaCDF	2.31	(2.25 – 2.37)	2.14	(0.00 – 13.50)
1,2,3,4,7,8-HexaCDF	0.24	(0.23 – 0.25)	0.23	(0.00 – 1.30)
1,2,3,6,7,8-HexaCDF	0.27	(0.26 – 0.27)	0.25	(0.00 – 1.10)
1,2,3,7,8,9-HexaCDF	0.00	(0.00 – 0.00)	0.00	(0.00 – 0.12)
2,3,4,6,7,8-HexaCDF	0.13	(0.13 – 0.14)	0.12	(0.00 – 1.10)
1,2,3,4,6,7,8-HeptaCDF	0.01	(0.01 – 0.01)	0.01	(0.00 – 0.17)
1,2,3,4,7,8,9-HeptaCDF	0.00	(0.00 – 0.00)	0.00	(0.00 – 0.01)
OctaCDF	0.00	(0.00 – 0.00)	0.00	(0.00 – 0.00)
**Coplanar-PCB (12 isomers)**	6.05	(5.88 – 6.23)	5.48	(0.07 – 35.19)
Non-Ortho3,3',4,4'-TetraCB	0.00	(0.00 – 0.00)	0.00	(0.00 – 0.03)
Non-Ortho3,4,4',5-TetraCB	0.00	(0.00 – 0.00)	0.00	(0.00 – 0.01)
Non-Ortho3,3',4,4',5-PentaCB	4.79	(4.64 – 4.95)	4.30	(0.00 – 28.00)
Non-Ortho3,3',4,4',5,5'-HexaCB	0.77	(0.75 – 0.79)	0.72	(0.00 – 4.50)
Mono-Ortho2,3,3',4,4'-PentaCB	0.06	(0.05 – 0.06)	0.05	(0.00 – 0.57)
Mono-Ortho2,3,4,4',5-PentaCB	0.02	(0.02 – 0.02)	0.01	(0.00 – 0.09)
Mono-Ortho2,3',4,4',5-PentaCB	0.27	(0.26 – 0.28)	0.24	(0.03 – 1.95)
Mono-Ortho2'3,4,4',5-PentaCB	0.00	(0.00 – 0.00)	0.00	(0.00 – 0.02)
Mono-Ortho2,3,3',4,4',5-HexaCB	0.09	(0.08 – 0.09)	0.08	(0.01 – 0.42)

Mono-Ortho2,3,3',4,4',5'-HexaCB	0.02	(	0.02	–	0.02	)	0.02	(	0.00	–	0.10)
Mono-Ortho2,3',4,4',5,5'-HexaCB	0.03	(	0.03	–	0.03	)	0.02	(	0.00	–	0.13)
Mono-Ortho2,3,3',4,4',5,5'-HeptaCB	0.01	(	0.01	–	0.01	)	0.01	(	0.00	–	0.03)

CI, confidence interval; Max, maximum; Min, minimum; PCB, polychlorinated biphenyl; PCDD, polychlorinated dibenzo-p-dioxin; PCDF, polychlorinated dibenzofuran; TEQ, toxic equivalence.

[a] Dioxin levels are presented as the World Health Organization TEQ (2005). Those less than the lower limit of quantitation were calculated as zero.

[b] Total dioxins = PCDDs (seven isomers) + PCDFs (10 isomers) + Co-PCBs (12 isomers)
